# Co-analysis in virtual spaces: engaging children with disabilities, families, and the community as research partners in low-resource settings

**DOI:** 10.1186/s40900-026-00892-7

**Published:** 2026-05-09

**Authors:** Karen S. Sagun, Paul Edward N. Muego, Maria Eliza R. Aguila

**Affiliations:** 1 College of Allied Medical Professions, University of the Philippines, Manila, Philippines; 2 College of Rehabilitation Sciences, University of Santo Tomas, Manila, Philippines; 3 College of Social Work and Community Development, University of the Philippines, Diliman, Philippines

**Keywords:** Co-analysis, Disability, Children, Philippines, Community engagement

## Abstract

**Background:**

Traditional disability studies often overlook children with disabilities, their families, and communities from analytical processes, particularly in low-resource settings. This methodological paper describes co-analysis conducted across virtual spaces with marginalized stakeholders as partners in data interpretation, examining whether participatory approaches can address epistemic injustices while navigating resource constraints in urban Philippine communities.

**Methods:**

Children were engaged in data-gathering through a mosaic approach (storytelling, drawing, photo elicitation, interview) across 24 individual virtual sessions, while adults participated in eight (8) online focus groups. We employed a four-pronged co-analysis approach with 49 participants from a community-based rehabilitation (CBR) program for urban low-resource communities: eight (8) children with disabilities aged 9–16 years, 18 parents, nine (9) CBR workers, and 14 local organization members. The co-analysis approach comprised: (1) participant-directed methodology, (2) grassroots epistemology, (3) iterative partnership building, and (4) collective reflexivity addressing researcher positionality.

**Results:**

Co-analysis revealed four insights. First, layered accessibility is essential for inclusion, combining universal design with disability-specific scaffolding. Second, co-analysis itself creates hierarchies as analytical frameworks privilege certain cognitive and communication abilities despite accessibility adaptations. We argue for epistemic pluralism, recognizing different ways of analytical contribution. Third, virtual spaces generated different hierarchies rather than eliminating them. Fourth, structural inequities persisted despite methodological innovation, as digital access, economic precarity, and cognitive demands reproduced exclusions our methodology aimed to dismantle.

**Conclusions:**

Achieving epistemic justice through co-analysis requires institutional resources, researcher humility that values diverse analytical contributions, and acknowledgment that participatory methods may expand access but cannot overcome structural inequalities without concurrent shifts in power and resources.

## Abstract (plain language summary)

Disability research has a long history of speaking about persons with disabilities rather than with them. Children with disabilities, their families, and communities are seldom engaged to interpret findings about their own lives, and this exclusion is most evident in communities where resources are scarce. In the Philippines, we worked with 49 participants from a community-based rehabilitation program in urban low-resource areas, including 8 children with disabilities (ages 9–16), 18 parents, nine (9) community workers, and 14 local organization members, as partners in understanding research data through online sessions. Children shared their experiences through storytelling, drawing, photography, and interviews across 24 individual sessions. Adults joined eight online group discussions. Instead of researchers deciding alone what the data meant, participants reviewed findings and told us what the information really meant to them. We learned four important lessons. First, making research accessible requires both a general design for everyone and specific support for different abilities. Second, even with our adaptations, children who could easily put their thoughts into words were more visible in the analytical process. But visibility is not the same as value. We believe all types of contributions should be valued equally. Third, online platforms reduced some barriers, like travel costs, but created new ones, such as poor internet access. Fourth, poverty, limited technology, and the demands of the process itself kept excluding some of the people we most wanted to include. This work reveals that better methods alone are not enough. Truly including marginalized voices demands shifts in power, resources, and who gets to decide what knowledge counts.

## Introduction

Globally, over 236.4 million children aged 0–17 years have moderate to severe disabilities, with the majority in low- and middle-income countries (LMIC), where access to early intervention and support remains limited [1, 2]. These children are disproportionately affected by poverty, exclusion from education, and restricted participation in everyday activities [3]. Yet, despite this scale, disability research has historically centered on adult experiences, only recently turning attention to the distinct realities of children and their families in under-resourced environments [4, 5]. Significant gaps persist. Current datasets frequently underrepresent or exclude these children with disabilities altogether, particularly those with communication challenges or severe impairments [6, 7] and research remains heavily concentrated in high-income countries, leaving LMIC contexts largely undocumented [8]. The result is a body of evidence that inadequately captures the lived experiences and outcomes of the very populations most affected. Addressing these gaps requires methodological approaches that move beyond conventional researcher-driven designs to genuinely include the voices of children with disabilities, their families, and communities in knowledge production.

## Epistemic justice and knowledge democracy

Epistemic justice, the recognition and fair treatment of individuals as knowers [9] has emerged as a critical lens for examining who produces knowledge in disability research and whose knowledge counts [10].

Fricker’s [11] foundational work identifies two interconnected forms of epistemic injustice. Testimonial injustice occurs when a person’s credibility is diminished due to bias. Hermeneutical injustice arises when dominant frameworks lack the interpretive resources for certain groups to articulate and validate their experiences. Dotson [12] extends this framework through the concept of contributory injustice, capturing how marginalized groups are systematically positioned as subjects of research rather than contributors to knowledge production. In disability research, these three forms of injustice do not operate in isolation. They compound one another, deepening the exclusion of children with disabilities and their families from shaping how their realities are understood.

Families of children with disabilities occupy a paradoxical epistemic position. Parents and caregivers hold deep observational knowledge of their children’s abilities, needs, and preferences. These are longitudinal insights that brief professional assessments cannot replicate. Yet this expertise is routinely discounted. Children with disabilities face systematic credibility deficits rooted in competence presumptions, or assumptions that they cannot reliably report on their own experiences [13]. Non-verbal forms of testimony are afforded less epistemic weight than spoken accounts [14]. Family caregivers, particularly mothers, encounter parallel testimonial injustices. Their insights are dismissed as emotionally compromised, with professionals labelling maternal concern as overprotective or irrational in ways that reflect gendered stereotypes [15]. Their longitudinal knowledge is discounted as merely anecdotal against the perceived objectivity of professional assessments. These biases are not incidental. They are structurally embedded within medical and educational institutions that privilege professional authority over experiential knowing, positioning both children with disabilities and their families as less credible witnesses to their own lives.

## The disability sector as co-researchers

Participatory research with individuals with disabilities as co-researchers has gained considerable momentum. Peer researcher training models and youth advisory groups across contexts such as Uganda, Kenya, and sub-Saharan Africa have demonstrated that shared ownership of research processes is achievable [16–18]. Participatory tools through photovoice and video have enabled data gathering that foregrounds participant perspectives [19, 20]. Yet inclusion in knowledge production remains uneven. Studies continue to gravitate toward participants considered easier to include, while those with complex impairments or intersecting marginalization are sidelined [21, 22] Limited resources frequently confine co-researcher involvement to data collection, with meaningful participation in analysis and dissemination phases rarely sustained [23]. Even when participatory designs are adopted, implementation often lacks the sustained engagement or genuine power-sharing that distinguishes partnership from consultation [16].

Power dynamics remain a persistent challenge. Several studies emphasize co-reflexivity, mutual respect, and shared decision-making as foundations for ethical co-analysis [24, 25] and participatory action research has shown potential for disrupting traditional hierarchies and fostering collective agency [26, 27]. However, imbalances between academic researchers and community partners persist, compounded by disparities among participants with differing disabilities or social positions [16, 25]. Existing frameworks often lack concrete guidance for navigating these dynamics, risking tokenistic participation that reinforces the very hierarchies participatory methods seek to dismantle [28].

A further tension lies in the analytical process itself. Participatory co-analysis can enhance validity by incorporating lived expertise into interpretation, producing richer and more grounded findings [24, 29]. Reflexive practices and iterative dialogue are considered essential for maintaining rigor and ethical integrity [20, 24, 30]. Yet a fundamental practical barrier remains. Academic researchers must train participants in analytical processes such as identifying patterns, abstracting from specifics, and organizing hierarchical categories [31]. This training demands substantial time, energy, and willingness to learn unfamiliar frameworks. Participants may lack time given competing life demands, may not wish to adopt academic analytical methods, or may engage differently with abstract thinking. The result is a paradox. Co-analysis aims to democratize knowledge production, yet requiring participants to work within researcher-defined analytical frameworks may inadvertently reproduce epistemic hierarchies by privileging academic ways of knowing. Conflicts between academic standards and community priorities further complicate collaboration [24, 28] while resource limitations and insufficient critical reflection on how positionality shapes interpretation can undermine the iterative processes that rigorous co-analysis demands [23, 25].

These challenges intensify when research involves multiply marginalized populations. Children with disabilities and their families in low-resource settings face the most acute constraints on training capacity, time availability, and access to resources, making the gap between participatory ideals and practice most visible where inclusive methods are most needed.

## Virtual methods as opportunity and challenge

The COVID-19 pandemic forced a rapid shift to digital platforms in participatory disability research, raising urgent questions about whether online methodologies could sustain meaningful participation by marginalized communities [32–34]. Virtual platforms offer tangible advantages. They reduce the need for in-person meetings, substantially address transportation barriers, and require lower costs [35]. For families of children with disabilities living in poverty, these benefits are not trivial. Yet the digital environment is far from neutral. It is a socially produced space shaped by the tools selected, the modes of interaction employed, and the engagement strategies adopted, all of which determine who can participate and on what terms [36, 37]. Platform design, connectivity requirements, and interface complexity create conditions that favor certain participants while marginalizing others. Drawing on this concept, we use virtual space as an analytical abstraction to capture the socially produced, unevenly distributed digital environments participants navigate. The plural virtual spaces signal heterogeneity across devices, connectivity conditions, household settings, and platforms.

This matters because the populations most likely to be excluded from digital research are precisely those whose perspectives are most needed. Children with disabilities, families living in poverty, and communities facing digital barriers hold knowledge that is essential to understanding the complexities of disability experiences, yet they are the least likely to have reliable internet access, appropriate devices, or environments conducive to sustained online engagement. Research that fails to account for these realities risks producing findings that reflect the experiences of the most digitally privileged rather than the most affected.

Recognizing these challenges, this methods paper explores participatory approaches that prioritize the voices of children with disabilities, their families, and communities with limited resources, examining how virtual co-analysis can be designed to expand inclusion while remaining critically attentive to the new forms of exclusion introduced by digital methods.

## Study context and aim

Community-based rehabilitation (CBR) is a development strategy designed to address barriers to services, access, and inclusion for children with disabilities in low-resource settings [38]. Active participation by stakeholders is a foundational principle [39]. Yet how participation is understood and practiced within CBR programs remains poorly documented, particularly in urban low-resource communities in the Philippines.

This gap shaped our research. We asked how children with disabilities, their families, CBR workers, and community organizations in urban low-resource communities understand and experience participation in CBR programs. Specifically, we sought to understand how different stakeholders define participation, what facilitates or hinders it, through what mechanisms it operates, and what outcomes it produces. However, the substantive findings about participation are reported in detail elsewhere. This paper focuses on the methodological innovation we employed to answer these questions.

Public involvement in research means carrying out research with or by members of the public rather than to, about, or for them [40]. We took this principle seriously. Children with disabilities, parents, CBR workers, and local organization members served as co-analysts rather than subjects. Their interpretations directly shaped categorical frameworks and research conclusions. The local organization led participant recruitment, and the research questions emerged from community-identified priorities about participation in CBR programs. The engagement process is described in Appendix A using the Guidance for Reporting Involvement of Patients and the Public-2 (GRIPP-2) Checklist [41].

This paper describes how we involved children with disabilities, their families, and their communities as co-analysts of data through virtual means, putting epistemic justice principles into practice while navigating digital divides and resource constraints in the Philippine context. We offer critical reflections on our layered accessibility framework, examine methodological tensions and differential participation across disability types, and propose recommendations for future participatory research in similar settings.

## Methodology

### Research design and study setting

This study combined participatory research principles [42, 43] with grounded theory analytical techniques [44, 45]. Each tradition served a specific purpose. Participatory principles ensured that stakeholders, including children with disabilities, their families, and community members, exercised authority over data interpretation. Grounded theory provided the systematic analytical structure needed to develop categories across multiple stakeholder groups while grounding those categories in community ways of knowing.

The study was conducted in Quezon City, the most populous city in the Philippines, with over 2.9 million inhabitants [46]. All participants were associated with the Quezon City Kabahagi Center for Children with Disabilities. The center’s CBR program, called “Kabahagi,” a Filipino word meaning “to be a part of,” has been operational since 2018 and provides healthcare, education, social, empowerment, and livelihood services to children with disabilities and their families from low-income backgrounds [47].

Ethical clearance was received from the University of the Philippines Manila Review Ethics Board (UPMREB 2022-0461-01). The study was conducted in accordance with the Declaration of Helsinki (2013 revision) and the National Ethical Guidelines for Health and Health-Related Research (NEGHHRR) 2017. Age-appropriate informed consent and assent procedures were followed for all participants, and both children and parents consented to the use of images and artwork for publication.

### Researcher positionality and reflexivity

The primary researcher (KS) is a Filipino occupational therapist serving as Director of the Quezon City Kabahagi program. This insider role established trusting relationships with participants but created power differentials, as KS manages services that families depended upon. To mitigate coercion, recruitment was conducted by a research assistant and a parent organization member, and the consent forms stated that participation would not affect program services. KS maintained a reflexivity journal documenting how her dual identity as service provider-researcher shaped analytical decisions. Co-researchers (PM and MA) are Filipino faculty in community development and physical therapy with no prior relationship to the program or participants. As outsiders, they provided critical distance, serving as auditors who systematically questioned whose voices were privileged and challenged KS’s interpretations throughout the analysis.

### Participants and recruitment

Participants were recruited through purposive sampling led by the Kabahagi Parent Advocates Organization, a civil society organization of over 500 parent members recognized by the city government. This community-led recruitment was deliberate. A parent member of the organization, supported by a research assistant, identified and invited potential participants, ensuring that the process was driven by the community rather than by researchers.

Eight children with disabilities participated in the study (Table 1). Inclusion criteria required that children had a disability, were enrolled in the CBR program, and resided in low-income urban communities. Parents provided informed consent before children were invited to give age-appropriate assent. The children ranged in age from 9 to 16 years and presented with diverse diagnoses and communication profiles, reflecting the heterogeneity of disability experiences within the program.

Adult participants comprised 18 parents of children with disabilities, 14 members and officers of the Kabahagi Parent Advocates Organization, and 9 CBR workers (Table 2). The sample was predominantly female across all groups. All organization members and officers were women, aged 35 to 67, while only one of the 18 parent participants was male, with age range of 23 to 58. This gender composition reflects broader patterns in caregiving and community rehabilitation work in the Philippines, where mothers disproportionately assume responsibility for disability-related care and advocacy.

Data were gathered from February to December 2023. It should be noted that, while 49 participants were recruited, co-analysis sessions involved shifting subsets of this sample across multiple rounds, as caregiving demands, connectivity challenges, and scheduling constraints led to variable attendance, particularly among parents and local organization members. This attrition is documented in the findings.

**Table 1:** Demographic profile of children with disabilities

Participants	Age	Sex	Diagnosis
Child 1	10	M	Intellectual Disability
Child 2	12	M	Intellectual Disability
Child 3	13	M	Attention Deficit Hyperactivity Disorder (ADHD)
Child 4	14	F	Focal Epilepsy
Child 5	16	M	Cerebral Palsy
Child 6	9	F	Klumpke’s Palsy
Child 7	14	M	Autism Spectrum Disorder
Child 8	12	M	Cerebral Palsy Spastic Focal Epilepsy

**Table 2:** Demographic profile of CBR workers

Staff No.	Designation	Sex	Age
1	Physical Therapist	M	27
2	Occupational Therapist	M	26
3	Audiologist	M	45
4	Speech Therapist	F	37
5	Occupational Therapist	F	26
6	Livelihood Coordinator	F	31
7	Occupational Therapist	F	26
8	Speech Therapist	F	49
9	Social and Empowerment Coordinator	M	40

### Data gathering methods

The mosaic approach provided a flexible, multi-modal framework for engaging the children with disabilities in data gathering. Designed as a universal approach, it offers multiple child-friendly data collection options that encompass different communication levels and modes of expression [42, 43]. Four methods were used across 24 individual online sessions with the eight children: storytelling, drawing, photo elicitation, and interview.

Each method served a distinct purpose. Storytelling, using narratives linked to research themes, prompted personal reflection and conversation. Drawing positioned children as the primary interpreters of their own artwork, giving them control over what they shared and how it was understood [48]. Photo elicitation offered a way for children to express thoughts when they are not confident in drawing, helping them feel more at ease in the research process [49]. Interviews provided opportunities for direct verbal interaction. Critically, children chose which modalities they preferred rather than having methods predetermined by their diagnosis. This was intentional. We wanted children to learn through experience that they held authority over how their knowledge would be expressed and at what pace [50].

In practice, children’s choices were often shaped by disability-related preferences and strengths. Children for whom verbal expression was not a primary communication mode, such as Child 1 with intellectual disability, primarily used drawing as their expressive mode. Children who were verbal and had physical disabilities, such as Child 5 with cerebral palsy, engaged more readily in interviews while parents assisted with photo elicitation activities. These variations illustrate that universal design does not mean uniform participation.

For adult participants, including parents of children with disabilities, local organization members, and CBR workers, online focus group discussions (FGDs) were used. FGDs allowed participants to build on each other’s responses, generating insights that individual interviews might not surface [51–53]. Groups ranged from seven to nine participants. Three focus groups were conducted with parents and three with local organization members, while two were conducted with CBR workers.

### Co-analysis approach

In our study, we employed a four-pronged co-analysis approach done virtually, attempting to actively engage children with disabilities, their families, and community stakeholders. We approached co-analysis as a negotiated process. Participant authority was meaningful and consequential, yet it operated within institutional, epistemic, and academic constraints that the methodology could partially redistribute but not fully dissolve. How these constraints shaped the analytical process is examined in the Limitations section.

### Participant-directed approach through flexibility

Participant direction and methodological flexibility were foundational to our co-analysis design. For children with disabilities, we employed a layered accessibility framework combining universal design with disability-responsive scaffolding.

The universal layer is applied to all children regardless of disability type. Findings were presented both visually and verbally, with children’s own drawings and photos used as anchors to guide discussion topics [54]. Child-friendly language was used throughout, with active friendliness as the facilitator’s mode. Children could accept, correct, or reject the researcher’s restatement of the concepts discussed, giving them direct authority over how their ideas were represented. Within this universal structure, specific disability-responsive scaffolds were tailored to each child’s communication and cognitive profile. Table 3 details the analytical skills required for co-analysis, the specific scaffolds implemented, and examples of how these operated in practice.

**Table 3:** Skills for co-analysis and scaffolding implemented

Skills needed for Co-Analysis	Scaffolds Implemented	Example
Verbal articulation of analytical insights	Yes/No questions or given multiple choices	Instead of clarifying a sense of connection as a relevant concept in participation, we asked, “Remember when you drew playing with friends? We think that’s about feeling connected to other people. Is that right?”
Categorical thinking	Concrete examples, grounded on their specific, remembered experiences.	Instead of clarifying happiness as a motivation for participation, we referred to answers based on experiences to probe into preliminary categories. For child 6, when she said she will plant trees relating to the storybook, we probed into this to ask “How do you feel when you plant trees for the community?”
Abstract social concepts	Concrete social scenarios, special interests integration as reflected in drawings or photos or narratives	Instead of clarifying about secure environment as essential in participation, we asked (in reference to the drawing), It says ‘rules.’ Why do they need to have rules there?

Parallel participant-directed structures governed adult stakeholder involvement. The local organization identified and recruited participants, recognizing their superior understanding of community members’ circumstances, trust levels, and readiness. Parents and CBR workers were explicitly invited across multiple sessions to propose additional questions, topics, or areas of concern they believed the research should address. This was an effort for the research to reflect community-identified priorities about what matters in their contexts.

By establishing participant direction as foundational from recruitment through analysis, we sought to build the relational trust that co-analysis demands. Whether we fully succeeded varied across participants, as the Results section examines.

### Grassroots epistemology

Our co-analysis process grounded interpretation in participants’ own ways of making sense of their experiences [55]. At the start of each subsequent session, initial findings were presented to all participant groups, creating an ongoing cycle of interpretation, challenge, and refinement. We presented preliminary findings verbally rather than in written documents, removing literacy as a barrier to engagement. Adult participants spoke in conversational Filipino, while children used English, Filipino, or both, allowing them to respond in whichever language felt most natural.

**Fig. 1:**
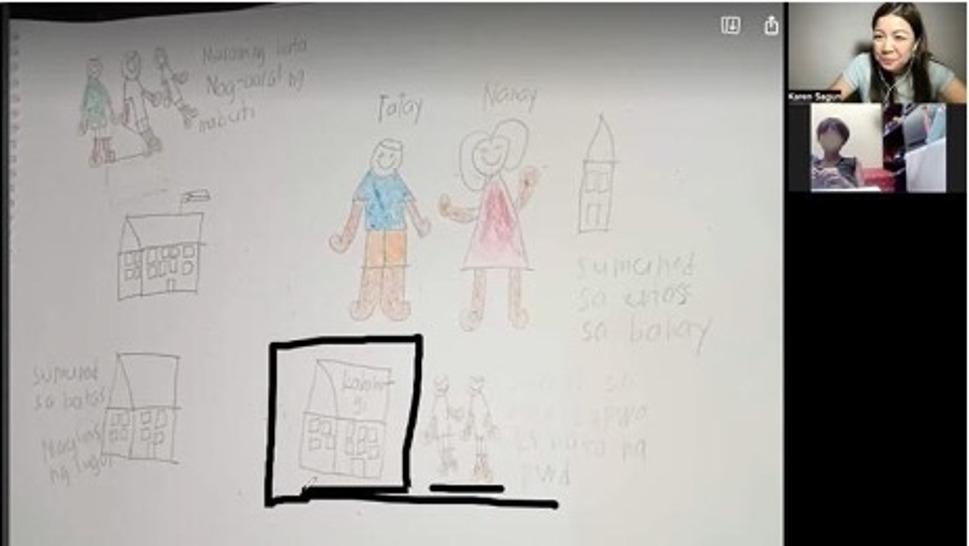
Drawings of a child shared on screen as illuminative work

For children, visual outputs were central. Drawings and photos were not subjected to researcher-led content analysis. Instead, children interpreted their own artwork, with the visual outputs functioning as “illuminative artwork” [56] (Fig. 1). This means the images served as communication tools through which children articulated meanings that the researcher could not have inferred independently. What a drawing meant was determined by the child who created it, not by the researcher who viewed it.

For parents of children with disabilities, representatives of local organizations, and CBR workers, their analytical contributions were integrated directly into evolving interpretations. Participant comments about preliminary findings were treated as data of equal weight to the original transcripts.

### Iterative partnership building

Building a genuine analytical partnership requires sustained engagement across multiple sessions, not a single consultation. Two power dynamics demanded ongoing attention throughout this process. The first was the relationship between the researcher and participants. The second was the conceptual interaction between adult and child participants, where adult interpretive authority risked overshadowing children’s contributions.

We remained reflexively attentive to how power structures across multiple social contexts shape how we, as academic researchers, “hear” what is said [57–59]. Each session functioned simultaneously as member checking and collaborative sense-making, with participants reviewing and building upon interpretations from previous sessions.

Co-analysis with participants with diverse cognitive abilities required extended timelines and repeated exposures. Children for whom abstract categorical thinking was already accessible engaged with it earlier in the process. Children with intellectual disabilities required multiple sessions before the analytical scaffolding enabled meaningful engagement with the concepts. We consider it a developmental trajectory of analytical engagement that demanded methodological commitment.

### Collective reflexivity

The primary author kept a reflexivity journal recording possible biases, analytical decisions, and the inherent tensions arising from her dual positionality as both the Director of the CBR program and the primary researcher. This insider-outsider position provided deep relational access and community trust built over time with children with disabilities and their families, yet simultaneously carried significant power differentials that could silence or distort participant voice. Recognizing that who conducts the analysis and the lens they bring profoundly impacts subsequent findings [60, 61], we implemented reflexivity as a group activity rather than a personal one, while acknowledging that this practice remained embedded within academic conventions that the research team ultimately controlled. Co-researchers (PM and MA) served as critical auditors who systematically interrogated whose voices were privileged throughout the analytical process and identified potential biases introduced by the primary researcher’s service-provider lens.

### Virtual spaces as means for co-analysis

Comprehensive technical support was implemented to prevent digital literacy from becoming a barrier to participation. Platform selection prioritized accessibility, familiarity, and screen-sharing capabilities. Participants received simple infographics explaining the process and direct one-on-one assistance before sessions began. For families sharing a single device, a common reality in urban low-resource households, sessions were scheduled around household availability and competing demands on the shared technology.

## Findings

### How co-analysis transformed research findings

The co-analytical process produced a deeper, more detailed understanding than a researcher-led study could have, highlighting that children with disabilities and their families have unique insights that should influence research outcomes [50, 55, 62]. These changes are evident in distinct stakeholder epistemologies that have emerged, reprioritized categories, a deeper understanding of visual and verbal data, and language shifts toward participant voice.

Initially, the research team’s preliminary analysis yielded seven (7) overarching categories that merged children’s and adult perspectives into a unified framework. However, through co-analysis sessions, it became evident that collapsing distinct stakeholder perspectives obscured critical differences in how participation was understood and experienced. Ongoing conversations showed that what researchers thought was thematic similarity actually indicated different ways of knowing. This led to the creation of six (6) categories from children and three (3) from adults, each with several subcategories. This distinction was not just about organization. It respected the idea that children’s understanding cannot be fully captured by adult views without losing important knowledge [50].

For example, children’s articulation of “child-friendly space” enhanced the adult’s category of “safe space” by foregrounding peer support as an enabling relational condition. Adult stakeholders highlighted the importance of physical access and freedom from discrimination, while children shared that spaces can be genuinely participatory only when peers actively welcome and support one another. This child-centered elaboration transformed “safe space” from a passive absence of threat into an active cultivation of peer solidarity.

Co-analysis revealed that participants prioritized findings differently from the researchers, assigning greater significance to categories the research team had initially treated as peripheral. Parents’ repeated emphasis on group dynamics during co-analysis sessions, for instance, signaled that internal cohesion and decision-making processes within the parent organization were not subsidiary concerns but central to their participation experiences. This shift showed that participants had the power to decide what was most important [55].

**Fig. 2:**
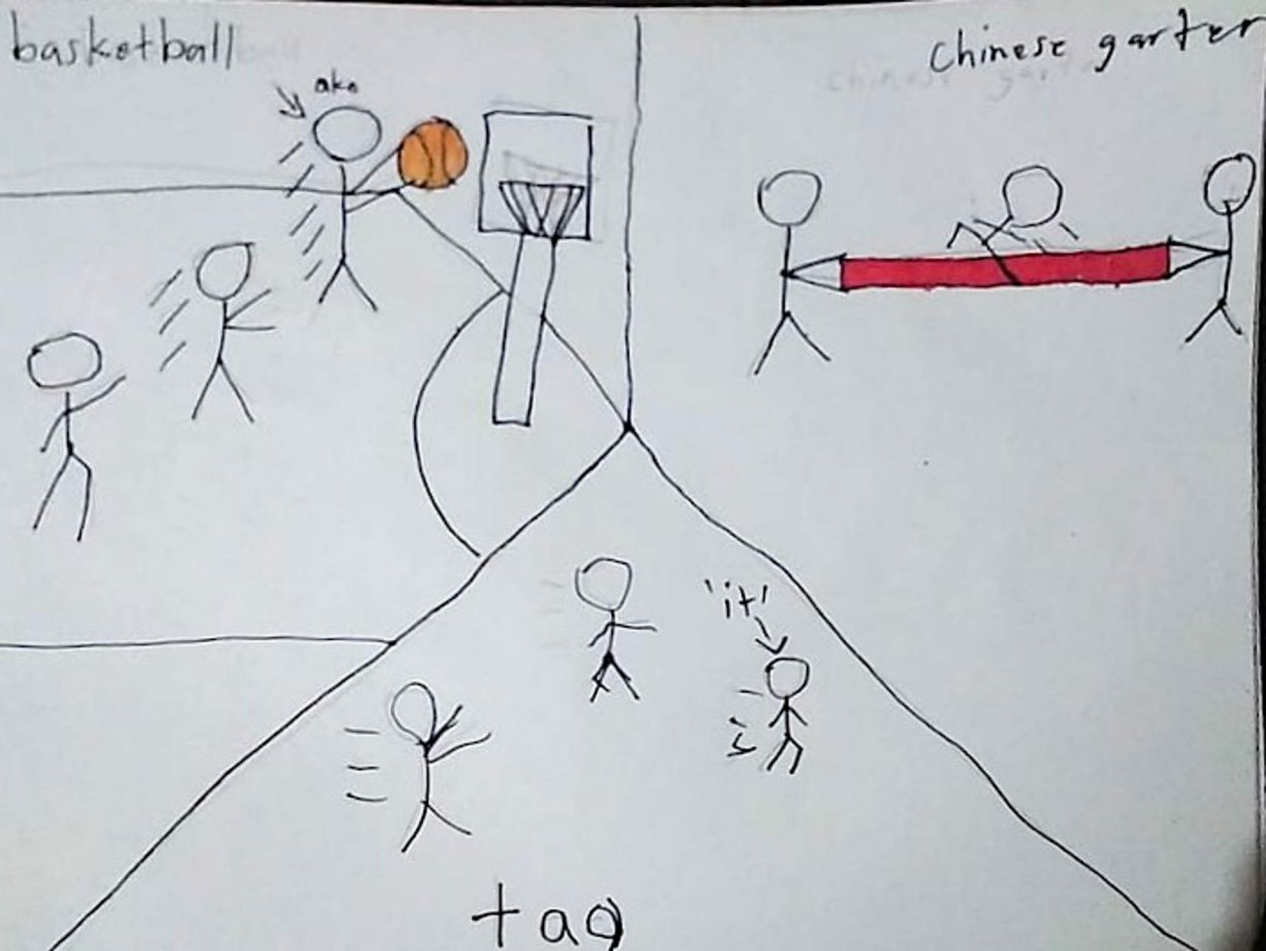
Drawing of play activities by Child 3

The co-analytical process also generated a deeper understanding of children’s meanings, particularly regarding visual data. For example, in Child 3’s drawing (Fig. 2), if relying solely on the researcher’s interpretation of the visual content, the drawing would have been coded simply as “playing with peers,” treating play as a discrete activity category.

However, when Child 3 interpreted his own drawing, he explained that playing facilitates socialization, enabling the formation of friendships and providing companionship. This reveals a causal chain linking individual activity to relational outcomes and community engagement that was invisible in the drawing itself. Child interpretations revealed that what appeared to be simple play actually represented children’s understanding of the necessity of social capital for community participation.

Lastly, the co-analysis led to significant changes in language that favored participants’ words over the researcher’s, especially in areas related to children’s experiences. Initial category names reflected the researcher’s framings, stemming from academic discourse and developmental paradigms. Through reflexive discussions, the research team expressed that these labels did not resonate with how the children actually conceptualized their experiences. Hence, the children’s categories were revised by adopting participants’ language and transforming them into a more experiential tone. For instance, the revision of the category from “participation as development” to “tools for participation” reflected children’s active understanding of themselves as deploying capabilities rather than passively undergoing developmental processes imposed by others.

### Analytical participation varied by skill demands

Children’s analytical participation varied according to three cognitive and communicative capacities inherent to co-analysis. The first capacity, verbal articulation of analytical insights, distinguished children who could independently generate verbal explanations from those whose analytical contributions took the form of experiential confirmation. Child 5 articulated elaborate insights about his building blocks: “Our gaining of knowledge provides fuel to think of new concepts… You can bring change not just to your community… but also to the entire country and the whole world.” In contrast, Child 2, when asked why people in his drawings were smiling, responded, “Because happy”. Probed further, he replied, “Because… it helps the community”. These can be considered as brief confirmations rather than elaborate explanations.

The second capacity, categorical thinking that abstracts from concrete experiences, distinguished children who identified patterns from those who validated when grounded in specifics. For example, Child 3 described causal patterns when he said, “I spoke about autism, then the effect was that they helped those with autism. I started a chain reaction.” The child abstracted from specific instances to broader categorical frameworks. On the other hand, Child 2’s interpretation of his drawing, labeled “Obey Orders at Home”, required concrete prompting about consequences, evident when he said, “My mother will get angry”, grounding in specific outcomes from experiences he can recall, rather than abstract patterns.

The third capacity, comprehension of abstract social concepts, revealed differential access to implicit social meanings. For example, Child 7 explained rules in child-friendly spaces when he said, “This ensures the space is always child-friendly… In order not to hurt someone’s feelings.” In contrast, Child 2 accessed concepts only through his drawings, which showed visible representations and required concrete anchors rather than engaging abstract constructs directly.

These three capacities created observable analytical hierarchies. Children for whom verbal fluency, categorical thinking, and social abstraction were accessible exercised the most visible analytical participation. The children independently generated insights, articulated mechanisms, and offered unprompted theoretical connections. Children for whom these capacities were less accessible participated through different modes of confirming, responding to prompts, and requiring scaffolding.

These hierarchies, however, describe differences in analytical visibility rather than analytical value. Experiential validation, affective insight, and confirmation through concrete anchoring are not lesser contributions that approximate the generative abstraction produced by more verbally fluent participants. They constitute distinct and irreplaceable epistemic inputs. Child 2’s confirmation that happiness was central to participation, grounded in lived experience rather than abstract reasoning, carried evidential weight that no amount of theoretical elaboration by other participants could replicate. Treating these contributions as equally valued, rather than as accommodated versions of a preferred analytical mode, is not a methodological concession but a prerequisite for genuine epistemic pluralism.

### Virtual experiences in co-analysis: affordances and barriers

The virtual spaces established multiple supportive conditions that made it easier for families to participate in research, especially those who would struggle with traditional in-person methods. The most important factor was the ease of joining from home without having to pay for transport or deal with geographical access, eliminating financial and physical obstacles that often impact low-income families with children who have disabilities. Moreover, the virtual format enabled participation during periods of public health risk when in-person gatherings would have exposed this vulnerable sector to infectious disease transmission.

Even with these advantages, the convergence of financial struggles, poor infrastructure, and lack of digital skills substantially challenged the implementation of virtual co-analysis and threatened to reproduce the epistemic exclusions the methodology sought to dismantle. Participants encountered persistent difficulties accessing reliable internet, with unstable signals leading to frequent disconnections. The suboptimal quality of internet service, characterized by low bandwidth and intermittent disruptions, occasionally compelled participants to relocate outside their homes to side alleys in search of stronger signals, causing them to miss critical portions of discussions and undermining their capacity to contribute fully to co-analysis. Most of the parents and local organization members joined the discussions from shared living spaces with limited privacy, where household members moved through the frame, addressed them directly, or created auditory distractions through television, conversations, or street noise from open windows. These environmental factors disrupted participants’ concentration and their ability to articulate complex interpretations coherently, fragmenting the collaborative sense-making process central to co-analysis.

Digital literacy issues made it even harder to access resources, as some parents lacked the technical skills to navigate online environments despite receiving pre-session simulations and real-time phone assistance. Login difficulties persisted across multiple sessions, attributed to password recall challenges and insufficient coordination of device use among households sharing a single device.

Frequent rescheduling was needed, particularly for mothers. Even with adjustments, maintaining consistent attendance at several joint analysis meetings was challenging. Only two of seven parent participants returned for the second focus group discussion, while five of nine participated in the third session. Similarly, among local organization officers and active members, only three of nine participants returned for the third discussion. This attrition meant that only a few participants could take part in the ongoing comparative analysis, as those who missed earlier meetings lacked the background needed to assess the changing category structures.

### Relational dynamics shaping participation

Establishing rapport and trust with participants proved more challenging in virtual contexts than in traditional face-to-face research, requiring deliberate strategies to create relational conditions conducive to co-analysis. The success of these methods became clear over time as children with disabilities showed more comfort and ownership. They displayed joy and pride when their past drawings were shown at the beginning of new sessions, with one child showing her artistic work, saying, “Teacher! Look at my drawing. It’s nice,” while another child showed excitement through wide eyes and a big smile. These reactions showed that presenting children with their earlier work built familiarity and continuity, boosting their engagement in the co-analytical process.

On the other hand, some sessions showed that children’s behavioral challenges increased due to background disturbances, which necessitated parents’ help to keep their attention. In effect, this also challenged the researcher in building rapport with the child. One child showed signs of losing interest, such as avoiding eye contact, yawning often, asking questions to be repeated, fidgeting, and showing frustration. This led to rescheduling the session to improve engagement. These situations highlighted the struggle between honoring children’s independence and research efficiency, with the team always placing children’s well-being and genuine involvement above set schedules.

Group interaction patterns during focus groups differed between parent groups and local organization members, reflecting underlying social hierarchies. Discussions with parents who were not organization members required extended warm-up periods, as participants initially felt uncomfortable and feared judgment from others. Although these discussions ultimately yielded rich data, they required substantial moderator prompting and active facilitation to draw out interpretations, suggesting that unfamiliarity among participants and with the research context constrained spontaneous co-analytical contributions.

## Discussion

This study makes three (3) methodological contributions to participatory disability research: (1) demonstrating that universal design requires disability-responsive scaffolds for genuine analytical access, (2) revealing that co-analysis itself contains cognitive and communication accessibility barriers requiring methodological pluralism, and (3) showing virtual methods create different rather than fewer participation barriers, with implications for epistemic justice.

### Layered accessibility for inclusion

This study aligns with current methodology research findings that universal design must be supplemented with additional supports to achieve inclusion [63]. Universal design, through the mosaic approach, substantially reduced expression barriers by offering modality choices. Children selected drawing or photography, storytelling, and interviews based on preference rather than diagnosis. This flexibility enabled diverse data collection. However, it did not automatically translate to accessible co-analysis. The transition from expressing experiences to interpreting their meanings presented distinct barriers that the mosaic approach cannot address [64]. Children who engaged enthusiastically in data gathering encountered different obstacles when analytical work required categorical thinking, abstract interpretation, and communication skills.

Disability-responsive scaffolds became essential at this analytical juncture. Concrete prompting grounded in children’s specific outputs, yes/no validation options, and the expression of emotions enabled analytical participation. These scaffolds addressed cognitive and communicative barriers that the mosaic approach alone could not eliminate. The distinction matters because current participatory literature often conflates accessible data collection with accessible analysis, assuming methods enabling children to express experiences automatically enable them to co-analyze meanings [65]. Our findings support that these constitute separate accessibility challenges requiring different supports.

This reveals that participation barriers operate at multiple levels, requiring differentiated responses. The practical implication is straightforward yet demanding. Participatory researchers must exhaust flexible methods at every research phase, not only in how children express knowledge but also in how they interpret it. However, the extent to which layered accessibility can achieve equity across cognitive diversity remains an open question, examined in the following section on how co-analysis itself privileges certain capacities.

### Co-analysis privileges cognitive and communication skills

The analytical hierarchies documented in the findings reflect differences in visibility, not value, a distinction with significant implications for how co-analysis is designed and evaluated. Co-analysis in itself privileges certain cognitive and communication abilities. Categorical abstraction, pattern identification, and expressive language skills are not equally accessible across participants. Yet this observation describes who was most analytically visible, not whose contributions mattered most. We argue that there are different forms of analytical contribution that are equally valuable. The experiential validation when Child 2 confirmed the home-to-community progression, or the affective analysis when the child drew smiling people contributing to emotional insight that happiness as central to participation is just as important to Child 5 and 7’s abstract analytical work. These contributions do not follow the traditional analytical framework, but profoundly shape the findings precisely because of their grounding in concrete experience. Recognizing diverse analytical modes as equally legitimate, rather than as accommodated approximations of a preferred generative abstraction, remains essential for genuine epistemic justice [65].

### Structural inequities persist despite methodological innovation

Digital technologies hold the potential to both mitigate and exacerbate epistemic injustices faced by children with disabilities and their families in urban low-resource communities. This dual potential is rooted in theories of epistemic injustice, which highlight the significance of social and infrastructural factors in accessing knowledge and participating in decision-making processes [66]. Providing mobile data credits, technical support, and flexible scheduling helped, but these were not sufficient. Virtual modalities risk creating new inequities through digital divides, leaving families with fewer resources in the shadows. The dropout trend in this participatory research highlights this issue, as ongoing analysis favors those who can consistently engage, potentially excluding invaluable perspectives from the most vulnerable families. This underscores that effective involvement requires not only cognitive ability but also equitable access to material conditions, such as time, space, and stability that poverty denies [36]. Gendered dimensions further exacerbate these challenges, as mothers often juggle unpaid care work alongside research involvement. Hence, virtual methods create different rather than fewer participation barriers. In the absence of structural supports, technology reproduces rather than resolves exclusion.

The relationships and power structures that developed across multiple sessions reveal both the potential and the vulnerability of knowledge-sharing partnerships. The shift from an initial hierarchy between researchers and participants to more equal relationships as co-analysts indicates that knowledge authority can be shared by openly demonstrating that participant insights genuinely influence the analysis. Participants felt their contributions were valued when they saw their words incorporated into evolving frameworks, which more effectively affirmed their authority [62].

The implications of co-analysis extend beyond immediate methodological outputs [10, 67]. It can significantly change the lives of participants, researchers, and the way we understand disability research. For participants, being recognized as authoritative knowers can be transformative. This epistemic recognition empowers individuals to generate consciousness-raising effects as they recognize patterns across experiences, potentially laying the groundwork for collective advocacy [68]. However, we need to carefully examine if these possible advantages are worth the significant challenges, like multiple sessions requiring ongoing dedication from families in unstable situations, the emotional effort of being involved, and the danger that being part of research may take more than it gives when the underlying conditions do not improve. For researchers, when participants question initial interpretations, it disrupts traditional authority and fosters a sense of humility in knowledge. This means understanding that having academic qualifications doesn’t automatically give one a better understanding of other people’s experiences. This humility should extend beyond personal reflection. It needs to influence the changes in institutions and fields about what is seen as expertise and who is acknowledged as a creator of knowledge [55]. These findings demonstrate that epistemic justice requires more than methodological innovation. Without structural transformation in terms of digital equity, economic security, and cognitive accessibility, participatory methods risk shifting burden of inclusion onto marginalized participants while leaving oppressive conditions intact.

## Limitations and implications

The intersectionality of disability type with poverty and age likely created distinct epistemic positions that our analysis did not fully capture. Our small sample size (*n* = 8 children) limited subgroup analysis, and our emphasis on individualized adaptations over categorical disability groupings reflected a tension between medical model categorization and social model emphasis on environmental barriers. Future research should explicitly examine how disability type, severity, and intersecting identities shape participatory research processes and the nature of knowledge produced. Additionally, digital gaps, dropout rates, and ongoing researcher control led to situations where certain voices influenced results more than others, even with a commitment to equitable involvement. The question of whether final analytical decisions remained with the research team despite participant influence, whether the insider-outsider positionality affected how views were interpreted, and whether the demands of participation meant taking from rather than empowering vulnerable families, all suggest that the boundary between genuine co-analysis and sophisticated consultation remains contested [69]. These are not methodological failures but inherent tensions in conducting participatory research within structural inequalities that individual studies cannot resolve. The uncertainty of how results might change with face-to-face co-analysis limits generalizability, while the urban poor Filipino community context also limits transferability.

Moving forward, these findings carry significant implications for disability research methodology and the operationalization of epistemic justice principles. Co-analysis must be recognized as an ethical imperative rather than an optional enhancement when research involves children with disabilities, their families, and communities, given that researcher-only interpretation constitutes epistemic injustice by excluding irreplaceable experiential knowledge [50]. However, genuine co-analysis demands institutional changes: funding mechanisms supporting extended timelines and multiple sessions, budgets for participant compensation, technical support, and infrastructure access, training in accessible methods, and ethical review processes facilitating community involvement. Virtual methods provide important access improvements but also lead to new barriers. This indicates that blended strategies and face-to-face interactions can effectively leverage the benefits of different methods while mitigating their drawbacks [70]. Future research should critically interrogate when participatory methods empower rather than burden marginalized populations. This study shows that challenges in low-resource environments are reasons to improve and further support participatory methods, not excuses for excluding knowledge. It emphasizes that the knowledge of children with disabilities and their families should be respected, especially when it means making significant changes to methods, investing resources, and showing humility as researchers. The challenge is not deciding whether we should seek epistemic justice through co-analysis, but rather figuring out how to achieve it in ways that truly shift power and resources, instead of merely shifting the burden of interpretation while maintaining existing structural inequalities.

## Conclusion

This study demonstrates that co-analysis with children with disabilities, their families, and communities is achievable in resource-constrained settings yet requires confronting critical methodological and institutional challenges. Accessibility necessitates layered approaches combining universal design foundations with disability-responsive scaffolds tailored to verbal, categorical, and social comprehension capacities. Epistemic pluralism emerges as essential. Experiential validation and affective insight constitute analytical contributions equal in value to abstract theoretical generation, though traditional analytical frameworks privilege the latter.

Achieving genuine epistemic partnership revealed tensions between methodological rigor and inclusive participation. Extended timelines and multiple sessions enabled deeper analysis but took labor from families navigating economic precarity and caregiving responsibilities. These findings suggest that epistemic justice extends beyond methodological adaptation to encompass institutional support and critical examination of whose cognitive capacities categorical frameworks privilege.

Virtual methods exemplified this broader challenge. They offered accessibility by eliminating transportation barriers, yet reproduced digital inequities, privileging participants with stable connectivity while marginalizing those most economically vulnerable. This pattern reveals a fundamental tension in participatory disability research. The challenge is not whether children with disabilities and their families possess analytical capacity. This study proves they do. Rather, the question is whether researchers and institutions will commit the resources and relinquish the control that genuine epistemic partnership requires, even when such commitments disrupt familiar research practices.

## Data Availability

Data sharing is not applicable to this methodological article.

## Abbreviations

CBR: Community- based rehabilitation
LMIC: Low- and middle- income countries
COVID-19: Coronavirus disease 2019
GRIPP-2 Checklist: Guidance for Reporting Involvement of Patients and the Public-2 Checklist
QC: Quezon City
FGD: Focus group discussion
